# Postprandial plasma glucose excursion is associated with an atherogenic lipid profile in individuals with type 2 diabetes mellitus: A cross-sectional study

**DOI:** 10.1371/journal.pone.0258771

**Published:** 2021-10-20

**Authors:** Po-Chung Cheng, Chia-Hung Kao

**Affiliations:** Graduate Institute of Biomedical Sciences, China Medical University, Taichung City, Taiwan; Universita degli Studi di Milano, ITALY

## Abstract

Coronary heart disease (CHD) is a prevalent complication of type 2 diabetes mellitus (T2DM). The atherogenic low-density lipoprotein (LDL) cholesterol is an established risk factor of cardiovascular disease, and evidence also suggests that postprandial plasma glucose (PPG) levels closely delineate CHD mortality in diabetes. The investigators hypothesized that postprandial plasma glucose excursion (PPGE), defined as the difference between 2-hour PPG and fasting plasma glucose (FPG), may be associated with plasma LDL cholesterol levels in patients with T2DM. This study enrolled diabetic participants for whom FPG and lipid profile were sampled after a 12-hour fast, followed by PPG sampling two hours after consuming a standard meal with 75 grams of carbohydrates. The study enrolled 379 participants who were divided into PPGE tertiles according to the difference between their 2-hour PPG and FPG. Participants in the highest PPGE tertile had considerably greater plasma LDL cholesterol levels than patients in the lowest tertile (126.7 mg/dL vs. 99.5 mg/dL, *P* <0.001). Linear regression analysis also demonstrated that the PPGE was positively correlated with plasma LDL cholesterol levels (β coefficient: 0.165, *P* < 0.001). Postprandial glucose excursion positively correlated with plasma LDL cholesterol levels in individuals with T2DM. Participants with raised PPGE harbored greater LDL cholesterol levels than those with lower postprandial glucose fluctuations. Therefore, postprandial glucose excursion is associated with an atherogenic lipid profile and may be a modifiable risk factor of diabetic CHD.

## Introduction

Type 2 diabetes mellitus (T2DM) is a metabolic disorder that induces substantial morbidity in affected patients [1]. Chronic hyperglycemia predisposes patients to microvascular complications including retinopathy, neuropathy, and kidney dysfunction [2]. Importantly, diabetes is also linked to macrovascular diseases such as coronary heart disease (CHD) and peripheral arterial disease [3]. Indeed, cardiovascular disease is a prevalent risk factor of mortality in T2DM [4], and prevention of diabetic CHD has therefore become an integral component of diabetes management guidelines [5,6].

Dyslipidemia in diabetes has several unique features. Insulin resistance increases the flux of free fatty acids to the liver, which subsequently produces an excess of very low-density lipoproteins, leading to elevated plasma triglycerides (TG), reduced high-density lipoprotein (HDL) cholesterol, and oxidized low-density lipoprotein (LDL) cholesterol [7]. Among these lipoprotein fractions, LDL cholesterol contributes to atheroma formation in blood vessels and is an established risk factor of CHD [8]. Therefore, plasma LDL cholesterol is a suitable therapeutic target for the prevention of cardiovascular disease in diabetes, with a level above 100 milligrams per deciliters considered supranormal in diabetic patients [9].

Compared to dyslipidemia, current evidence suggests that plasma glucose plays a less important role in the progression of diabetic CHD. As observed in the United Kingdom Prospective Diabetes Study, for each 4% elevation in serum glycosylated hemoglobin (HbA_1c_), there was a corresponding 10-fold increase in the incidence of retinopathy but only a twofold rise in CHD incidence [10]. Moreover, correlation studies demonstrated that a considerable proportion of diabetic complications could not be explained by changes in serum HbA_1c_ [11], implying that serum HbA_1c_ alone may not capture the entire spectrum of hyperglycemic complications.

Recently, plasma glucose variability after meals has gained attention as a potential risk factor of cardiovascular complications. In the Whitehall study, the risk of CHD increased in line with plasma glucose levels after an oral glucose tolerance test [12]. Moreover, a European research consortium observed that 2-hour postprandial plasma glucose (PPG) levels closely correlated with cardiovascular risk in T2DM [13]. Therefore, several investigators have proposed that postprandial hyperglycemia may be an HbA_1c_-independent risk factor of diabetic CHD [14].

Considering the link between postprandial hyperglycemia and cardiovascular risk, the investigators hypothesized that postprandial plasma glucose excursion (PPGE), defined as the difference between 2-hour PPG and fasting plasma glucose (FPG) levels, may influence the plasma lipid profile in a hyperglycemic environment. This study investigates the relationship between PPGE and plasma LDL cholesterol levels in patients with T2DM. Moreover, additional clinical determinants of plasma LDL cholesterol levels in diabetic participants will be examined.

## Materials and methods

### Study population

Participants in this cross-sectional study were recruited from Changhua Christian Hospital in Taiwan. Candidates visiting the Endocrinology clinic between June 2013 and May 2016 were screened for eligibility. Inclusion criteria to participate in this study were as follows: (1) patients between 21 and 90 years of age, (2) clinically established diagnosis of T2DM according to the criteria of the American Diabetes Association [5], including serum HbA1c level ≧6.5%, FPG ≧ 126 mg/dL, 2-hour plasma glucose levels after 75 gram oral glucose tolerance test ≧ 200 mg/dL or random plasma glucose levels ≧ 200 mg/dL with classic symptoms of diabetes, (3) recipients of metformin monotherapy for diabetes, (4) recipients of at least six months of statin medications, and (5) compliance with blood tests and dietary instructions.

Exclusion criteria were as follows: (1) patients with familial hypercholesterolemia, chronic kidney disease, hypothyroidism, or hemoglobin disorders, (2) recipients of antidiabetic drugs other than metformin, (3) recipients of lipid-lowering therapies other than statin, (4) history of chronic alcoholism, (5) recipients of estrogen replacement therapy, (6) recipients of antidepressants, (7) pregnancy and (8) patients undergoing chemotherapy or radiotherapy for cancer treatment.

### Demographic information

Baseline demographic data including age, sex, body weight and height were recorded at the clinic visit. Six months prior to the blood tests, participants received comprehensive medical nutrition therapy according to current guidelines. Specifically, patients were instructed to limit saturated fat to less than 5% of total calories and increase whole grain intake. This adjustment aimed to address the potential confounding effect of dietary habits on plasma lipid levels. Dosage of metformin and statins were adjusted according to patients’ tolerance of medication side effects.

The presence of metabolic syndrome in this study was defined using the U.S. National Cholesterol Education Program criteria, with waist circumference thresholds modified for Asian adults, that requires at least three of the following: (1) waist circumference ≥ 90 cm for males or ≥ 80 cm for females, (2) plasma TG ≥ 150 mg/dl, (3) plasma HDL cholesterol < 40 mg/dL for males or < 50 mg/dL for females, (4) blood pressure ≥ 130/85 mm Hg or treated for hypertension, or (5) fasting plasma glucose 110 mg/dl or treated for diabetes [5].

### Laboratory evaluation

Participants underwent a 12-hour fast during which all medications were withheld prior to blood sampling for plasma TG, LDL cholesterol, HDL cholesterol, FPG, serum creatinine, serum alanine transaminase, serum high sensitivity C reactive protein (hs-CRP), and serum HbA_1c_. Thereafter participants consumed a standard meal, which consisted of 75 grams of carbohydrates, 5 grams of fat and 10 grams of protein, under the supervision of diabetes educators. Two hours after the meal, venous sampling for PPG level was performed. Blood samples were delivered to the central laboratory within one hour of venous sampling and assayed by Beckman Coulter UniCel DxC 800 Synchron Clinical Systems. The analytical precision was within 1.7 mg/dL for HDL cholesterol, within 3.0 mg/dL for LDL cholesterol, within 7.5 mg/dL for TG, and within 2.0 mg/dL for plasma glucose level. The plasma glucose level was quantified by the Hexokinase-UV/NAD end reaction method, whereas plasma HDL cholesterol, LDL cholesterol and TG were measured by the timed endpoint method using a commercial polyanion solution.

### Ethics approval

This study was conducted in accordance to the World Medical Association’s Declaration of Helsinki. The protocol was approved by the Institutional Review Board of Changhua Christian Hospital (IRB identifier: Y_108_0013). Participants provided informed consent to take part in the study.

### Statistical analysis

Participants were divided into tertiles according to their PPGE, defined as the difference between 2-hour PPG and FPG. Power calculation suggests that a sample size of 105 participants in each tertile is necessary to attain 80% statistical power. The Kolmogorov–Smirnov test was used to verify that clinical variables were normally distributed. All parameters in this study were normally distributed except for plasma triglycerides levels, and the Box-Cox procedure was used to normalize plasma triglyceride levels for statistical analysis. Baseline characteristics including age, gender, mean serum HbA_1c_, and plasma lipid fractions were compared between PPGE tertiles using one-way analysis of variance with post hoc Tukey’s test. Furthermore, a linear regression analysis was performed to assess the influence of clinical variables on plasma LDL cholesterol levels. Finally, binary logistic regression and sensitivity analysis were performed to verify the link between PPGE and LDL cholesterol. The Hanley-Mcneil method was used to compare the performance of area under the curve (AUC) of different models with PPGE, without PPGE, with remnant cholesterol, and with ratio of triglycerides and remnant cholesterol. Statistical analysis was performed using Statistical Package for the Social Sciences (version 22.0, SPSS, Chicago, IL), with a two-tailed *P* value of less than 0.05 interpreted as statistically significant.

## Results

**Fig 1:**
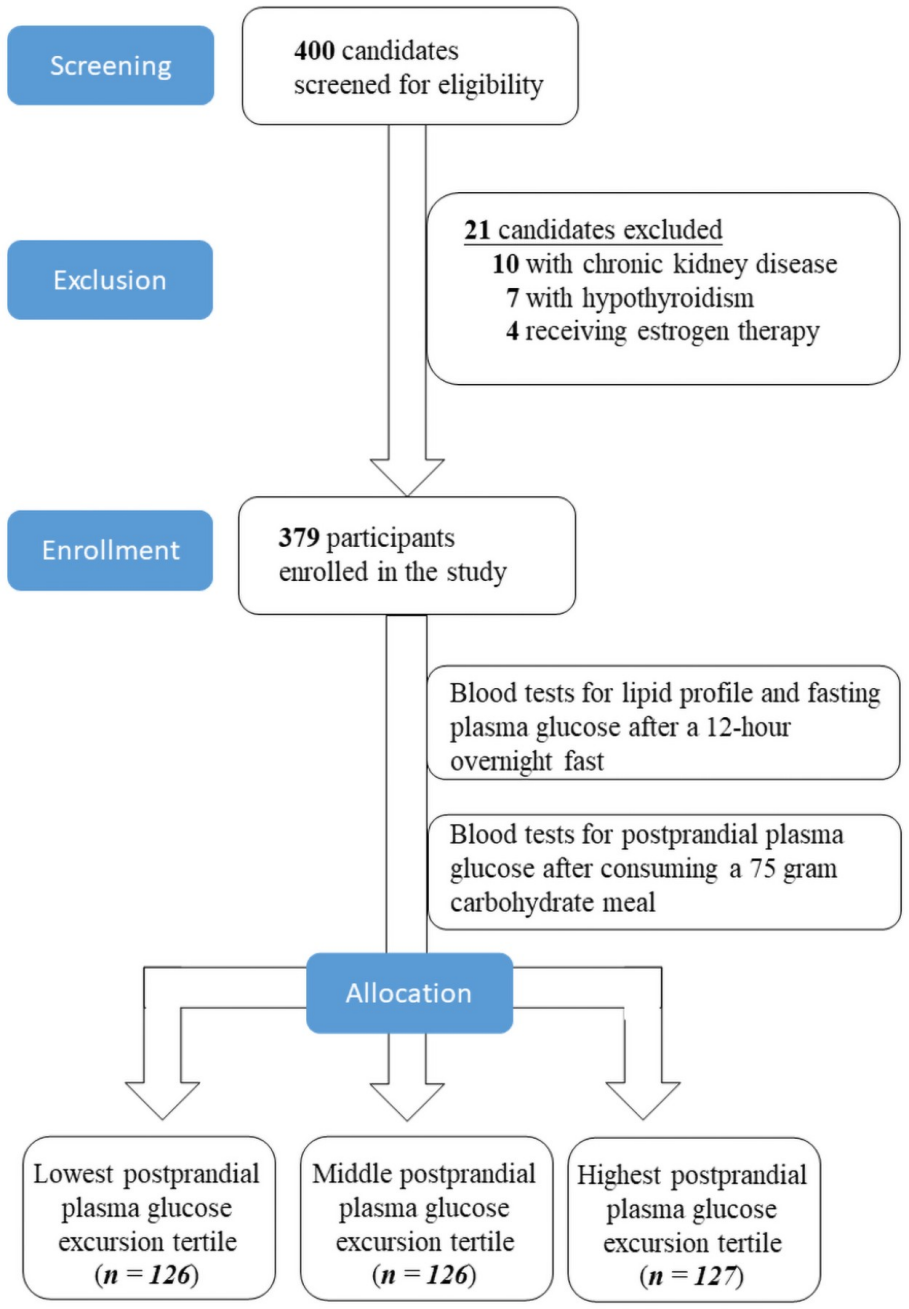
Enrolment protocol of the study.

This study screened 400 patients visiting the Endocrinology clinic for eligibility. Ten individuals were excluded due to chronic kidney disease, seven patients were ineligible due to coexisting hypothyroidism, and four people were excluded for receiving estrogen replacement therapy. The enrolment protocol is illustrated below (Fig 1).

### Demographic characteristics of the participants with association between PPGE and plasma lipid profile

The study enrolled 379 participants who were divided into tertiles according to their PPGE. As demonstrated in Table 1, demographic characteristics of the participants including age, sex, serum HbA1c, body mass index, blood pressure, serum creatinine, duration of diabetes, proportions with the metabolic syndrome, waist circumference, and serum alanine transaminase were similar between PPGE tertiles. Moreover, a similar proportion of patients in the PPGE tertiles received either rosuvastatin 10 mg, atorvastatin 20 mg, and pravastatin 40 mg, which are equivalent moderate-intensity statins [9]. Patients in each tertile also received similar dose of metformin per day. In the context of plasma glucose levels, participants had comparable FPG levels but considerably different 2-hour PPG levels, which established the basis of this investigation.

**Table 1:** Demographic features of the participants.

Parameters	Lowest PPGE tertile (*n* = 126)	Middle PPGE tertile (*n* = 126)	Highest PPGE tertile (*n* = 127)	*P* value
Age (years)	62.0 ± 11.6	61.0 ± 12.9	59.7 ± 13.4	0.368
Sex (Female)	74 (58.7%)	58 (46.0%)	69 (54.3%)	0.122
Serum HbA_1c_ (%)	8.12 ± 1.88	8.56 ± 2.21	8.46 ± 2.05	0.201
Diabetes duration (years)	5.8 ± 3.0	5.1 ± 3.5	6.0 ± 3.2	0.208
Proportions with metabolic syndrome (number, %)	19 (15.1%)	22 (17.4%)	23 (18.1%)	0.522
Waist circumference (cm)	61.1 ± 11.7	62.6 ± 13.5	61.0 ± 11.9	0.532
BMI (kg/m^2^)	26.2 ± 4.28	26.3 ± 4.74	26.0 ± 4.72	0.889
SBP (mm Hg)	131 ± 16.4	131 ± 15.8	133 ± 16.8	0.628
DBP (mm Hg)	78.3 ± 11.6	79.6 ± 10.1	78.6 ± 10.7	0.596
Serum creatinine (mg/dL)	0.826 ± 0.233	0.876 ± 0.259	0.839 ± 0.224	0.232
Serum ALT (U/L)	33.9 ± 21.7	35.8 ± 26.0	35.7 ± 27.9	0.788
FPG (mg/dL)	122 ± 22.0	128 ± 32.1	130 ± 34.3	0.086
2-hour PPG (mg/dL)	134 ± 22.7	169 ± 35.8	242 ± 63.7	<0.001
PPGE (mg/dL)	11.8 ± 6.04	40.9± 11.1^a^	111.8±55.3^a**,**b^	<0.001
Use of medications				
Rosuvastatin 10 mg	48 (38.1%)	42 (33.3%)	37 (29.1%)	0.578
Atorvastatin 20 mg	52 (41.3%)	60 (47.6%)	61 (48.0%)	0.506
Pravastatin 40 mg	26 (20.6%)	24 (19.0%)	29 (22.8%)	0.888
Metformin dose (mg per day)	1310 **±** 468	1313 **±** 472	1374 **±** 504	0.491
Plasma total cholesterol (mg/dL)	175.8 ± 37.9	189.5 ± 39.6^a^	202.7 ± 36.0^a,b^	< 0.001
Plasma TG (mg/dL)	156.6 ± 97.5	149.1 ± 71.4	142.9 ± 72.9	0.408
Plasma HDL-C (mg/dL)	45.0 ± 11.64	44.9 ± 11.3	47.8 ± 12.3	0.080
Plasma LDL-C (mg/dL)	99.5 ± 29.4	114.5±33.4^a^	126.7±28.2^a,b^	<0.001
Plasma non-HDL-C (mg/dL)	130.8 ± 35.5	144.5 ± 37.3 ^a^	155.1 ± 33.4 ^a,b^	<0.001
Plasma remnant cholesterol (mg/dL)	31.2 ± 19.5	29.6 ± 14.2	28.8 ± 14.7	0.462
Serum hs-CRP (mg/dL)	0.2 ± 0.13	0.25 ± 0.19	0.28 ± 0.2^a^	0.0022

Data are expressed as means with standard deviation of the mean for continuous variables and number (%) for categorical variables. Variables are compared between groups using one-way analysis of variance for continuous variables and Pearson’s χ^2^-test for categorical variables. Postprandial plasma glucose excursion is defined as the difference between 2-hour postprandial and fasting plasma glucose levels. ALT: Alanine aminotransferase, BMI: Body mass index, FPG: Fasting plasma glucose, PPG: Postprandial plasma glucose, SBP: Systolic blood pressure, DBP: Diastolic blood pressure, HbA_1c_: Glycosylated hemoglobin A_1c_, PPGE: Postprandial plasma glucose excursion, mg/dL: Milligrams per deciliter, mm Hg: Millimeters of mercury, kg: Kilograms, m: Meters, mg: Milligrams, hs-CRP: High sensitivity C reactive protein, TG: Triglycerides, HDL-C: High density lipoprotein cholesterol, LDL-C: Low density lipoprotein cholesterol.

As established in the study design, mean PPGE of the highest tertile was significantly greater than that of the lowest tertile (112 mg/dL vs. 11.8 mg/dL, *P* < 0.001). Participants in both the highest and lowest PPGE tertiles had similar levels of plasma TG (142.9 mg/dL vs. 156.6 mg/dL, *P* = 0.408) and HDL cholesterol (47.8 mg/dL vs. 45 mg/dL, *P* = 0.08). However, patients in the highest PPGE tertile had substantially greater levels of plasma LDL cholesterol (126.7 mg/dL vs. 99.5 mg/dL, *P* <0.001), total cholesterol (202.7 mg/dL vs. 175.8 mg/dL, *P* < 0.001), as well as non-HDL cholesterol (155.1 mg/dL vs. 130.8 mg/dL, P < 0.001) than those in the lowest PPGE tertile. Moreover, patients with the highest PPGE harbored higher serum hs-CRP levels relative to those in the lowest PPGE tertile (0.28 mg/dL vs. 0.2 mg/dL, P = 0.0022). These findings are also summarized in Table 1.

### Clinical determinants of plasma low-density lipoprotein cholesterol levels

As shown in Table 2, linear regression analysis demonstrated that the PPGE was positively correlated with plasma LDL cholesterol levels (β coefficient: 0.165, *P* < 0.001). In contrast, other clinical parameters did not demonstrate an association with plasma LDL cholesterol levels in this study.

**Table 2:** Linear regression analysis of parameters associated with plasma low-density lipoprotein cholesterol concentration.

Parameters	β coefficient	*P* value
Age (years)	-0.21	0.12
BMI (kg/m^2^)	0.62	0.1
Serum creatinine (mg/dL)	-0.24	0.45
SBP (mm Hg)	-0.1	0.3
Diabetes duration (years)	0.68	0.17
Serum ALT (U/L)	-0.11	0.1
FPG (mg/dL)	-0.08	0.15
Serum HbA1c	0.52	0.52
PPGE (mg/dL)	0.16	< 0.001

Postprandial plasma glucose excursion is defined as the difference between 2-hour postprandial and fasting plasma glucose levels. BMI: Body mass index, FPG: Fasting plasma glucose, Alt: Alanine aminotransferase, HbA_1c_: Glycosylated hemoglobin A_1c_, PPGE: Postprandial plasma glucose excursion, mg/dL: Milligrams per deciliter, mm Hg: Mmillimeters of mercury, kg: Kilograms, m: Meters, mg: Milligrams.

### Binary logistic regression and sensitivity analysis of plasma low-density lipoprotein cholesterol prediction

**Fig 2:**
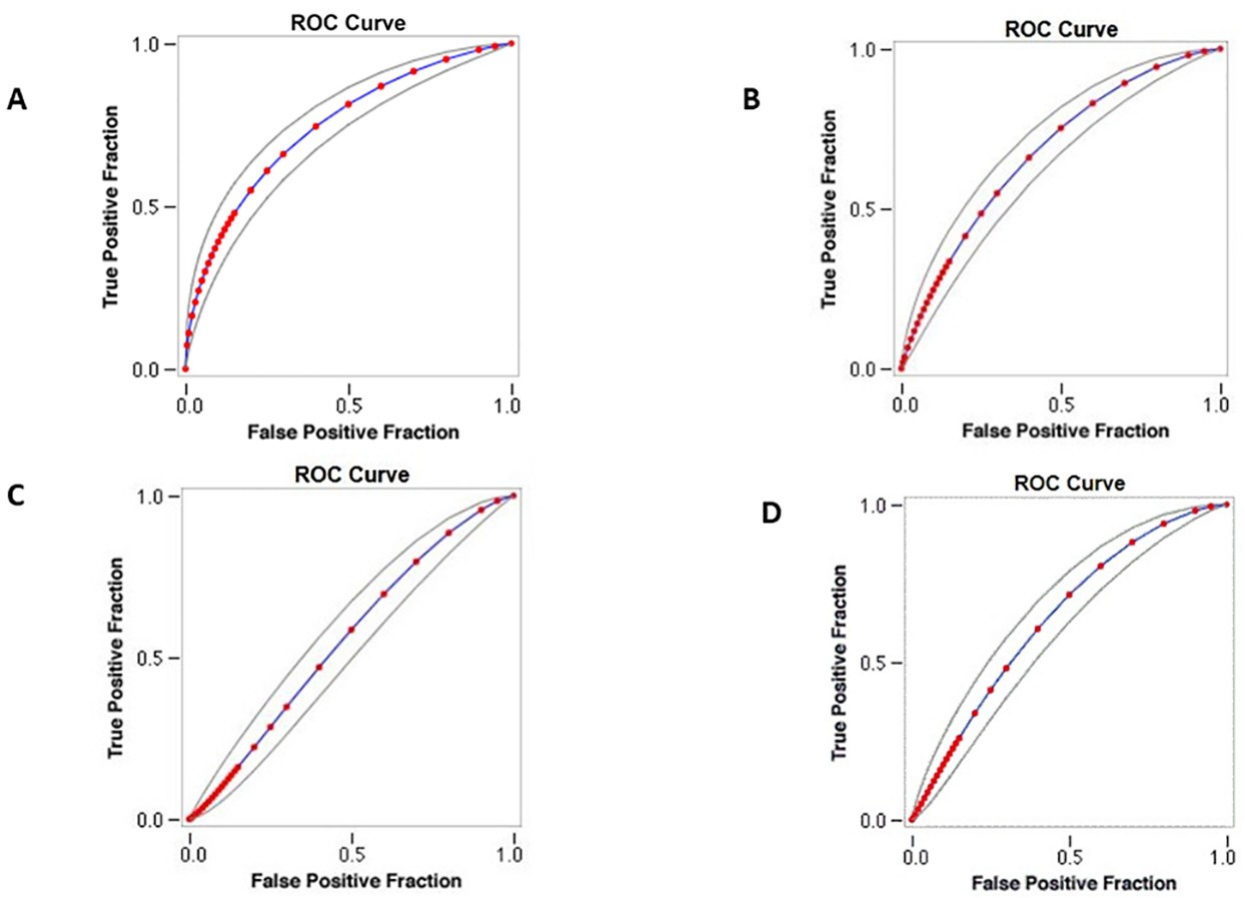
Sensitivity analysis with receiver operating characteristics curve. (A) Model with postprandial glucose excursion. (B) Model without postprandial glucose excursion. (C) Model with plasma remnant cholesterol. (D) Model with stratification by ratio of triglycerides and remnant cholesterol.

Binary logistic regression analysis verified an independent link between PPGE and LDL cholesterol levels (Coefficient: 0.125, P < 0.001). Sensitivity analysis with receiver operating characteristics curve, as shown in Fig 2A, demonstrated that PPGE is correlated with LDL cholesterol levels with an AUC of 0.744. In contrast, sensitivity analysis showed an AUC of 0.68 for the model without PPGE and an AUC of 0.62 for the model with remnant cholesterol, as shown in Fig 2B and 2C respectively. A model that stratifies participants by ratio of triglycerides and remnant cholesterol levels demonstrates an AUC of 0.64, as shown in Fig 2D.

Application of the Hanley-Mcneil method showed a difference in AUC between models with PPGE and without PPGE (Δ AUC: 0.06, standard error (SE): 0.036, *P* = 0.03), with PPGE and remnant cholesterol (Δ AUC: 0.12, SE: 0.04, *P* = 0.001), and with PPGE and ratio of triglycerides and remnant cholesterol (Δ AUC: 0.1, SE: 0.038, *P* = 0.0035).

## Discussion

This study observed a positive correlation between postprandial glucose excursion and LDL cholesterol levels in patients with T2DM. Participants with higher PPGE had greater plasma LDL cholesterol levels than patients with lower postprandial glucose fluctuations. Moreover, linear regression analysis verified that postprandial hyperglycemia significantly correlated with plasma LDL cholesterol levels. Sensitivity analysis demonstrates that PPGE has an appreciable correlation with LDL cholesterol levels in diabetic participants. Nonetheless, further studies are necessary to confirm this association in different patient populations.

Postprandial hyperglycemia may promote the accumulation of atherogenic LDL cholesterol through several pathways. Transient hyperglycemia accelerates the glycation of LDL cholesterol particles, which impairs their receptor-mediated catabolism by the liver [15]. Glycation also prolongs the half-life of LDL cholesterol and enhances its oxidation to form atherogenic metabolites [16]. Recent studies also demonstrated that transient hyperglycemia induces epigenetic changes in cholesterol receptors of the liver [17], leading to dysfunctional lipid catabolism [18]. Moreover, postprandial hyperglycemia promotes the generation of reactive oxygen species that impair the clearance of circulating lipid metabolites [19], in addition to micro-ribonucleic acid particles that disrupt the endothelial function of coronary arteries [20,21]. Recent evidence also suggests that transient hyperglycemia disrupts coronary flow reserve in patients with T2DM [22,23].

The observation in this study that PPGE positively correlated with plasma LDL cholesterol levels has clinical implications. Antidiabetic medications that target postprandial hyperglycemia such as alpha-glucosidase inhibitors (AGI) and glucagon-like peptide 1 (GLP-1) agonists can lower cardiovascular event rates in T2DM, partly through reduction of plasma LDL cholesterol levels [24,25]. In terms of CHD prevention, therefore, patients with raised PPGE may benefit from antidiabetic medications that target postprandial glucose variability, which may also reduce plasma LDL cholesterol levels. In addition to pharmacologic interventions, medical nutrition therapy has been shown to alleviate postprandial hyperglycemia [26], which potentially attenuates the atherogenic lipid profile in individuals with raised PPGE. At-risk individuals may also benefit from regular physical exercise to mitigate postprandial glucose fluctuations [27]. Moreover, this study demonstrated that participants with similar levels of serum HbA_1c_ may nonetheless have different PPGE. This finding concurs with the NHANES III study, which has shown that in diabetic patients with similar levels of serum HbA_1c_, nearly 40% demonstrated substantial postprandial hyperglycemia while the remainder had normal postprandial glucose levels [28].

The strength of this study includes a sizeable patient population enrolled from our endocrinology clinic, which renders the findings applicable to general practice. Participants withheld all medications during the 12-hour fast before blood tests to reduce potential confounding effects of these medications on plasma glucose levels and lipid profile. Moreover, certified diabetes educators supervised the study to avert potential errors in plasma glucose sampling and provided comprehensive medical nutrition therapy to participants to ensure similar levels of dietary lipid intake.

Nonetheless, this study has several limitations. Firstly, PPGE was assessed by consuming a standard meal and may not reflect daily glycemic excursion. The use of continuous glucose monitoring may more accurately reflect PPGE. Moreover, additional factors that influence postprandial glucose levels, such as the metabolic syndrome or insulin resistance, were unaccounted for in the study protocol. Importantly, secretory reserve of the pancreas can modify postprandial glucose levels but insulin levels were not quantified in the study. Furthermore, since postprandial glucose determination is influenced by concomitant hyperlipidemia, plasma lipid levels at the time of laboratory testing may interfere with the accuracy of PPGE quantification. Although postprandial lipid profile has been shown to correlate with cardiovascular risk [29], many clinical laboratories, including the one at the investigators’ institution, require lipid sampling under fasting condition to avoid postprandial variability in plasma TG [30]. Therefore, the absence of postprandial lipid profile in this study constitutes an additional limitation. Importantly, since participants in this study received statins in accordance with diabetes management guidelines [5], the findings in the study are applicable only to diabetic patients treated with statins.

## Conclusions

In conclusion, postprandial glucose excursion positively correlated with plasma LDL cholesterol levels in individuals with T2DM. Participants with raised PPGE harbored greater LDL cholesterol levels than those with lower postprandial glucose fluctuations. Therefore, postprandial glucose excursion is associated with an atherogenic lipid profile and may be a modifiable risk factor of diabetic CHD.

## Supporting information

S1 File Research dataset for the study. (XLSX)
